# An evaluation of purified *Salmonella* Typhi protein antigens for the serological diagnosis of acute typhoid fever

**DOI:** 10.1016/j.jinf.2017.05.007

**Published:** 2017-08

**Authors:** Nga Tran Vu Thieu, Tan Trinh Van, Anh Tran Tuan, Elizabeth J. Klemm, Chau Nguyen Ngoc Minh, Phat Voong Vinh, Duy Pham Thanh, Thanh Ho Ngoc Dan, Trung Pham Duc, Pinky Langat, Laura B. Martin, Jorge Galan, Li Liang, Philip L. Felgner, D. Huw Davies, Hanna K. de Jong, Rapeephan R. Maude, Masako Fukushima, Lalith Wijedoru, Aniruddha Ghose, Rasheda Samad, Arjen M. Dondorp, Abul Faiz, Thomas C. Darton, Andrew J. Pollard, Guy E. Thwaites, Gordon Dougan, Christopher M. Parry, Stephen Baker

**Affiliations:** a The Hospital for Tropical Diseases, Wellcome Trust Major Overseas Programme, Oxford University Clinical Research Unit, Ho Chi Minh City, Viet Nam; b The Wellcome Trust Sanger Institute, Hinxton, Cambridgeshire, United Kingdom; c Sclavo Berhing Vaccines Institute for Global Health, Siena, Italy; d Department of Microbial Pathogenesis, Yale University School of Medicine, New Haven, CT, USA; e Department of Medicine, Division of Infectious Diseases, University of California, Irvine, CA, USA; f Department of Internal Medicine, Division of Infectious Diseases and Center for Infection and Immunity Amsterdam (CINIMA), Academic Medical Center, University of Amsterdam, Amsterdam, The Netherlands; g Mahidol-Oxford Tropical Medicine Research Unit (MORU), Faculty of Tropical Medicine, Mahidol University, Bangkok, Thailand; h Centre for Tropical Medicine and Global Health, Oxford University, Oxford, United Kingdom; i Clinical Sciences, Liverpool School of Tropical Medicine, Liverpool, United Kingdom; j Chittagong Medical College Hospital, Chittagong, Bangladesh; k Malaria Research Group and Dev Care Foundation, Bangladesh; l Sheffield Teaching Hospitals NHS Trust Foundation and the University of Sheffield, Sheffield, United Kingdom; m Oxford Vaccine Group, Department of Paediatrics, University of Oxford and the NIHR Oxford Biomedical Research Centre, Oxford, United Kingdom; n The Department of Medicine, University of Cambridge, Cambridge, United Kingdom

**Keywords:** Typhoid fever, Enteric fever, *Salmonella* Typhi, Diagnostics, Bangladesh, Vi polysaccharide, IgM, Febrile disease

## Abstract

**Objectives:**

The diagnosis of typhoid fever is a challenge. Aiming to develop a typhoid diagnostic we measured antibody responses against *Salmonella* Typhi (*S*. Typhi) protein antigens and the Vi polysaccharide in a cohort of Bangladeshi febrile patients.

**Methods:**

IgM against 12 purified antigens and the Vi polysaccharide was measured by ELISA in plasma from patients with confirmed typhoid fever (n = 32), other confirmed infections (n = 17), and healthy controls (n = 40). ELISAs with the most specific antigens were performed on plasma from 243 patients with undiagnosed febrile disease.

**Results:**

IgM against the *S*. Typhi protein antigens correlated with each other (rho > 0.8), but not against Vi (rho < 0.6). Typhoid patients exhibited higher IgM against 11/12 protein antigens and Vi than healthy controls and those with other infections. Vi, PilL, and CdtB exhibited the greatest sensitivity and specificity. Specificity and sensitivity was improved when Vi was combined with a protein antigen, generating sensitivities and specificities of 0.80 and >0.85, respectively. Applying a dynamic cut-off to patients with undiagnosed febrile disease suggested that 34–58% had an IgM response indicative of typhoid.

**Conclusions:**

We evaluated the diagnostic potential of several *S*. Typhi antigens; our assays give good sensitivity and specificity, but require further assessment in differing patient populations.

## Introduction

Enteric (typhoid) fever is a systemic infection caused by *Salmonella enterica* serovars Typhi (*S*. Typhi) and Paratyphi A (*S*. Paratyphi A).^1^, ^2^ There are an estimated 12 million cases of typhoid (*S*. Typhi only) worldwide annually leading to approximately 120,000 deaths.^3^, ^4^ The organisms are transmitted via the fecal-oral route and the disease remains common in low/middle income countries in South/Southeast Asia and sub-Saharan Africa.^5^ Despite *S*. Paratyphi A being an emergent cause of enteric fever in parts of South and Southeast Asia,^6^ *S*. Typhi remains the most commonly reported etiological agent of enteric fever in Asia and Africa.

Typhoid occurs only in humans, making it a disease that can technically be eradicated.^7^ Indeed, typhoid has all but been eliminated from several countries in Southeast Asia where it was the most common cause of hospitalized febrile disease 20–30 years ago.^8^, ^9^ Elimination in these areas is generally attributed to extensive improvements in sanitation rather than widespread immunization schemes. The lack of data regarding the long-term impact of mass immunization for typhoid and the performance of licensed vaccines have hindered immunization as a sustainable typhoid control and elimination strategy. Future considerations for rational control measures for typhoid will rely on more accurately assessing disease burden, which requires a reliable diagnostic approach.^10^

All commonly used typhoid diagnostics perform poorly and are a roadblock for disease control efforts.^11^, ^12^ Currently, the only reliable method for the identification of febrile individuals with typhoid is the culture of a causative organism from a biological specimen.^12^, ^13^ However, this procedure is restricted to laboratories with adequate equipment and microbiology training, and the method has a limited sensitivity due to low concentrations of organisms in the peripheral circulation.^14^, ^15^, ^16^ Low bacterial loads have a similar impact on other methods that rely on detecting the presence of the infecting organisms, such as antigen detection or nucleic acid amplification. These methods are often reported to be highly sensitive, but have unrealistic performances; pre-treatment with antimicrobials is likely to compound this issue further.^11^, ^15^ New typhoid diagnostics are a necessity and various approaches have been evaluated, including measurement of innate immune responses,^17^, ^18^ antibody in lymphocyte supernatants,^19^, ^20^ and the identification of metabolomic signatures.^21^ However, these advances are still restricted to research laboratories and are not yet ready to be developed into simple, rapid diagnostic tests (RDTs).

We previously exploited a protein microarray to identify a multitude of immunogenic *S*. Typhi protein antigens to which an antibody response was generated during the early stages of typhoid.^22^ With the aim of validating antigens that could be used in a diagnostic assay we expressed and purified several of these potentially serodiagnostic *S*. Typhi antigens and investigated their diagnostic performance in a cohort of febrile Bangladeshi patients.

## Materials and methods

### Ethical approval

The study was conducted according to the principles expressed in the Declaration of Helsinki. The Bangladesh National Research Ethical Committee (BMRC/NREC/2010-2013/1543), the Chittagong Medical College Hospital Ethical Committee, the Oxford Tropical Research Ethics Committee (OXTREC 53-09) gave ethical approval for the study. Informed written or thumbprint consent was taken from the subject, their parent or caretaker for all enrollees.

### Study site, population and study design

The site and recruitment for this study has been descried previously.^23^ Briefly, Chittagong Medical College Hospital is a 1000-bed hospital serving Chittagong and the surrounding province. Adults and children (>6 months) consecutively admitted from January to June 2012 to the adult and pediatric wards at CMCH with an axillary temperature of ≥38 °C up to 48 h after admission and history of fever for <2 weeks were eligible for the study.

Here the gold standards for typhoid diagnosis were and blood culture and PCR amplification from blood using a previously described method.^15^ Blood (5–12 mL for adults and 1–2 mL for children) was cultured using Bact/Alert-FA and PF blood culture bottles, bottles were incubated in the Bact/Alert automated system (Biomerieux, France) for five days. The patient demographics and diagnostic testing results for this study are reported elsewhere.^23^ For the purposes of this investigation plasma samples from 40 healthy adult control subjects (hospital staff at the study site), 32 cases of confirmed typhoid (16 cases confirmed by blood culture, 13 cases confirmed by PCR and three cases confirmed by both blood culture and PCR), 17 cases from patients with confirmed febrile diseases other than enteric fever (*Staphylococcus aureus* (2), *Streptococcus pneumoniae* (1), *Streptococcus acidominimus* (1), *Enterococcus gallinarum* (1), *Escherichia coli* (2), *Klebsiella pneumoniae* (1), *Enterobacter cloacae* (2), *Acinetobacter* spp. (1), *Burkholderia cepacia* (3), dengue (2), *R. typhi* (2) and *O. tsutsugamushi* (1)) and 243 febrile patients with undiagnosed febrile disease were subjected to serological assays.

### PCR amplification, gene expression and protein purification

We selected 18 *S*. Typhi antigens that gave a differential serodiagnostic signal using protein microarray screening for further expression and purification (Table S1). The coding sequences of the selected genes, excluding trans-membrane domains were PCR amplified from CT18 genomic DNA and cloned into the 5′ *Nco*I and 3′*Not*I restriction sites of pET28b(+) vector (Novagen, UK) for further His-Tag purification. *E. coli* DH5α were transformed with the plasmid constructs for stable storage and *E. coli* BL21(DE3)pLysS (Promega, WI, USA) were used for expression and purification (Table S1).

For protein expression, the *E. coli* BL21(DE3)pLysS strains harboring unique plasmid constructs (pEK90-pEK109) containing the genes of interest were inoculated into LB broth containing 100 mg/L kanamycin (Sigma, MO, USA), and incubated at 37 °C overnight. Overnight cultures were diluted (1:100) into LB broth and incubated at 37 °C with agitation until optical density (OD_600_) of 0.5. Expression of the exogenous proteins was induced by the addition of isopropyl-β-D-thiogalactoside (IPTG) (Sigma–Aldrich, UK), to a final concentration of 0.1 mM. Bacterial cells were harvested (5000 × *g* at 4 °C for 10 min) after 3 h of incubation at 24 °C.

For soluble proteins, bacterial pellets were resuspended in 50 mM phosphate buffer (pH 8) containing 300 mM NaCl and 10 mM imidazole. After sonication, cell debris and the membrane fragment were pelleted by centrifugation at 16,000 × *g* at 4 °C for 30 min. Supernatants were filtered through a 0.45 μm membrane before being rocked at 4 °C with nickel coated agarose beads (Ni-NTA, Invitrogen) for 2 h. Protein bound Ni-NTA beads were loaded into gravity flow columns (Qiagen, Germany) and washed with 20 mM imidazole in phosphate buffer. Proteins were eluted with 250 mM imidazole in phosphate buffer. For insoluble proteins a denaturing protocol was performed by firstly incubating the bacterial cells in an 8 M urea (pH 7.8) solution containing 20 mM sodium phosphate and 500 mM NaCl. Proteins were eluted with 4 M Urea (pH3) in a solution containing 20 mM Sodium Phosphate buffer and 500 mM NaCl. Proteins were renatured after purification in 50 mM Sodium Phosphate solution and 500 mM NaCl.

### ELISAs using *S*. Typhi protein antigens

ELISAs to detect antigen specific IgM in human plasma samples were performed as described previously with 12 purified protein antigens and *S*. Typhi Vi polysaccharide antigen.^24^, ^25^ Briefly, 96 well flat-bottom ELISA plates (Nunc 2404, Thermo Scientific) were coated overnight with 100 μl per well of the various antigens (final concentrations; 7 μg/ml of protein antigens and 1 μg/ml for the Vi antigen in 50 mM Carbonate Bicarbonate buffer). Coated plates were washed and blocked with 5% milk solution in PBS. After 2 h of blocking, plates were washed and incubated with 100 μl (per well) of a 1:200 dilution of plasma at ambient temperature for 2 h. Plates were washed again and incubated with 100 μl per well of alkaline phosphatase-conjugated anti-human IgM at ambient temperature for 1 h. The final ELISA plates were developed using p-Nitrophenyl phosphate (SigmaFAST N1891, Sigma–Aldrich, UK) substrate for 30 min at ambient temperature and the final absorbance were read at dual wavelengths (405 nm and 490 nm) using an automated microplate reader (Biorad). End point positive absorbance results were defined as optical densities (OD) greater than the absorbance obtained for the blank control wells plus four times the standard deviation. Three wells of culture *S*. Typhi positive plasma were run as control for every 96-well plate for each antigen. The results of each ELISA plate were accepted only if the OD values of the controlled were within their range of their known values plus/minus two standard deviations of the blank wells.

### Statistical analysis

A geometric mean optical density was calculated to summarize the IgM response to the *S*. Typhi antigens in each arm of validation group including the negative reference population samples (healthy controls and other confirmed febrile infections) and the positive reference population (febrile patients with confirmed *S*. Typhi). The Wilcoxon signed-rank test was used to test the null hypothesis; no difference in optical densities between the patient groups. Spearman's rho was used to investigate potential correlations between IgM antibody responses against the various antigens. Receiver operating characteristic (ROC) curves were used to determine the optimal cut-off and the specificity and sensitivity of the various antigens. A performance estimation of more than one antigen combination was evaluated using Support Vector Machine (SVM). All analyses were performed with R software (version 3.3.1; R Foundation for Statistical Computing). All confidence intervals (CIs) are reported 2-sided at the 95% intervals; all other significant testing were performed 2-sided with a significance level of *p* < 0.05.

## Results

### Acute IgM antibody responses against *Salmonella* Typhi antigens

Of the 18 protein antigens we targeted to purify, we were able to express and purify 12 (Table 1).

**Table 1:** *Salmonella* Typhi antigens expressed in this study for serological testing.

Gene ID number^a^	Isotype detected using array^b^	Gene name	Amino acid identity *S*. Typhi CT18/*S*. Paratyphi A AKU_12601	Annotation
STY0452	IgM	*yajI*	163/165 (98%)	Putative lipoprotein – Prokaryotic homolog of protein DJ-1
STY0796	IgM	*ybgF*	260/262 (99%)	Putative exported protein – Tol/pal system protein
STY1086	IgM	–	187/187 (100%)	Putative lipoprotein
STY1372	IgM	*pspB*	74/74 (100%)	Phage shock protein B
STY1612	IgM	–	Absent	Putative membrane protein – prophage associated
STY4539	IgM	*pilL*	Absent	Putative exported protein – type IV pili
STY1522	IgG	–	292/296 (99%)	Putative secreted protein – Choloylglycine secreted homolog
STY1703	IgG	*ssaP*	123/124 (99%)	Putative secreted protein – T3SS
STY1767	IgG	*nlpC*	48/140 (34%)	Putative lipoprotein – Endopeptidase
STY1886	IgG	*cdtB*	268/269 (99%)	Cytolethal distending toxin subunit B homolog
STY3208	IgG	–	277/279 (99%)	Hypothetical protein – Unknown function
STY4190	IgG	*yhjJ*	190/192 (99.0%)	Putative Zinc-protease

a Parkhill et al. 2001.^48^

b Liang et al. 2013.^22^

**Figure 1:**
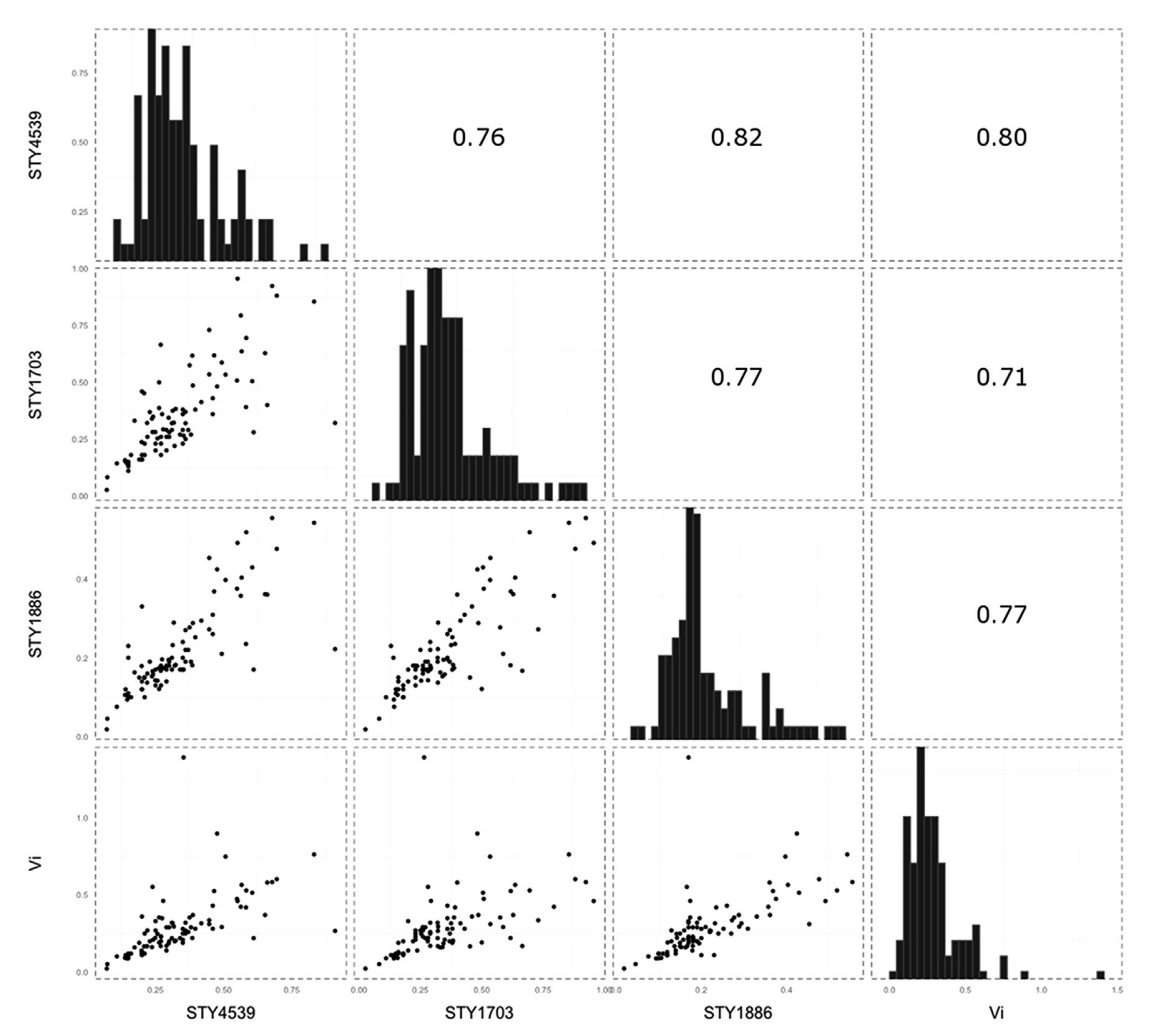
Correlation of IgM measurements between selected *Salmonella* Typhi antigens. A representative selection of data showing a correlation in IgM measurements in human plasma for the antigens encoded by STY4539, STY1703, STY1886, and the Vi antigen. Histograms show the distribution of IgM levels by optical density to the highlighted *S*. Typhi antigen. The scatterplots below the histograms plot the IgM measurements of the two antigens on a right angle to the histograms and describe the correlation between antibody responses to two selected antigens. The numerals above the histograms depict the Spearman correlation coefficient (rho) values of the mirrored scatterplot. All other correlations are shown in Fig. S1.

**Fig. S1:**
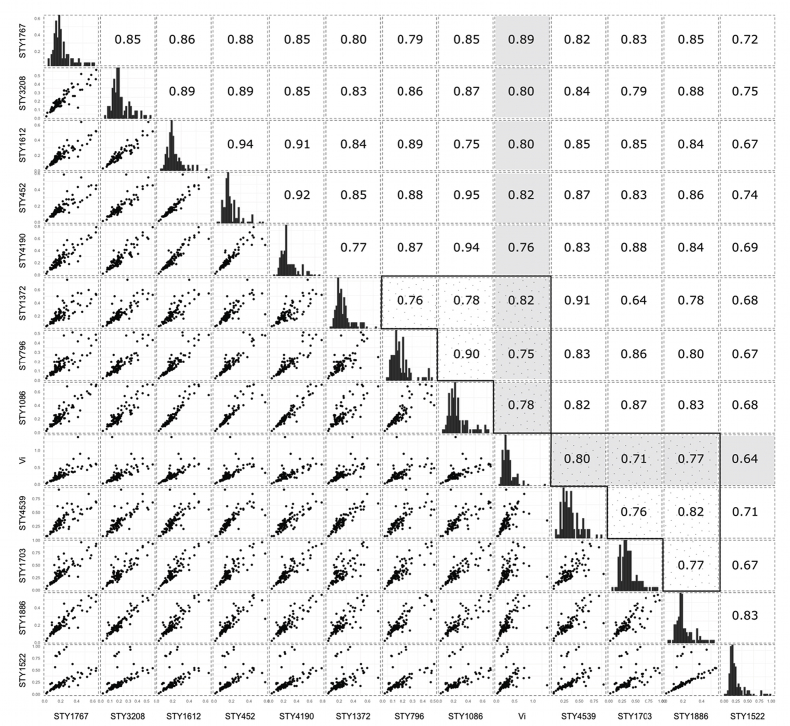
Correlation of IgM measurements between *Salmonella* Typhi antigens. Histograms show the distribution of IgM levels by optical density to all purified *S*. Typhi antigens. The scatterplots below the histograms plot the IgM measurements of the two antigens on a right angle to the histograms and describe the correlation between antibody responses to two selected antigens. The numerals above the histograms depict the Spearman correlation coefficient (rho) values of the mirrored scatterplot.

We firstly performed ELISAs independently employing the purified protein antigens and the Vi polysaccharide to detect IgM in the plasma of 40 healthy control subjects, 17 febrile individuals with a confirmed infection other than typhoid, and 32 individuals with either blood culture or PCR (or both) confirmed typhoid infections (n = 89 samples). We measured detectable IgM against all twelve of the purified *S*. Typhi proteins and the Vi polysaccharide in all of the 89 samples subjected to ELISA. We compared the IgM titers from each of the antigens individually to assess the performance of the antigens and to identify potential correlations between the serological targets. We found that early IgM responses against the majority of the novel *S*. Typhi protein antigens, with the exception of STY1522 (rho < 0.7), were highly correlated with one other (rho > 0.8). Notably, the IgM response to the protein antigens correlated weakly with those directed against the Vi polysaccharide (rho < 0.6) (Fig. 1 and Fig. S1).

### The diagnostic potential of IgM against *Salmonella* Typhi antigens

**Figure 2:**
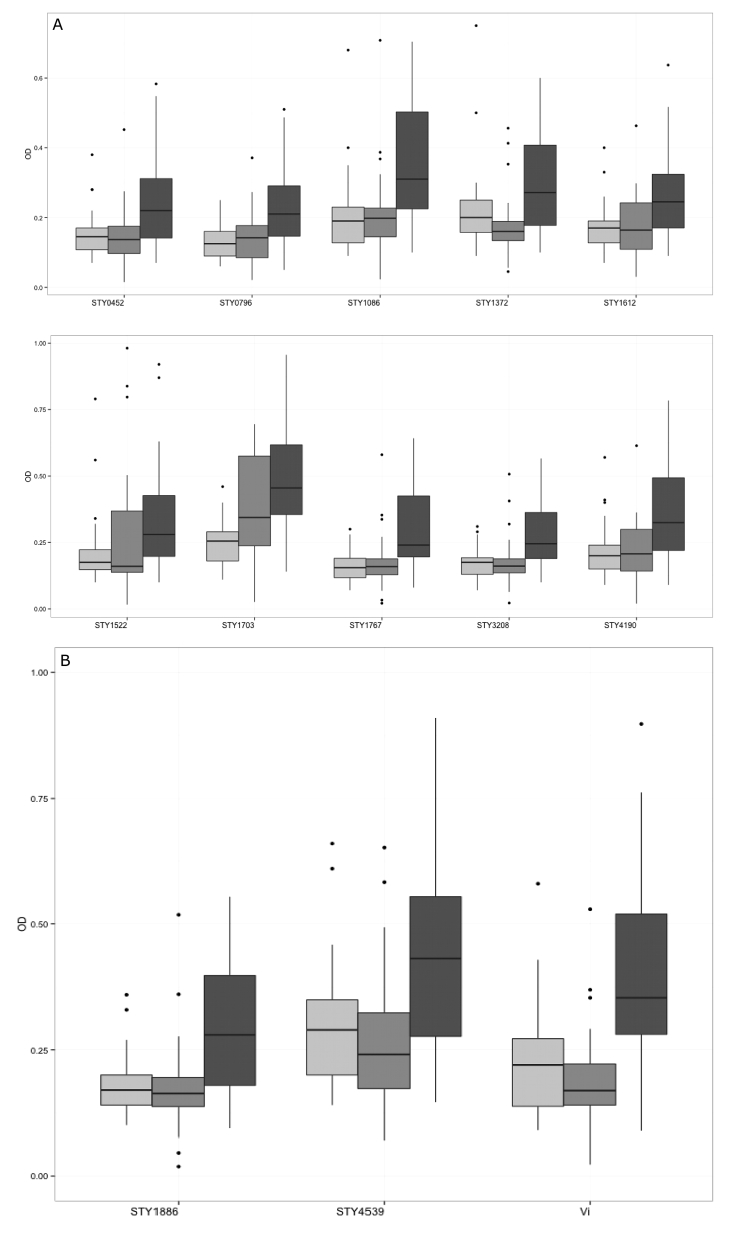
IgM responses against *Salmonella* Typhi antigens in a Bangladeshi cohort of febrile patients and controls. Boxplots showing IgM measurements (optical density) in plasma from afebrile controls (light gray), febrile patients with an infection other than typhoid fever (medium gray), and confirmed typhoid patients (dark gray). Dark horizontal lines represent the mean IgM measurement, with the box representing the 25th and 75th percentiles, whiskers represent the 5th and 95th percentiles; outliers are represented by dots. A) Boxplots of antibody responses against (from left to right and upper to lower) STY0452, STY0769, STY1086, STY1372, STY1612, STY1522, STY1703, STY1767, STY3208, and STY4190. All mean antibody measurements were statistically significant between the healthy controls and typhoid infections and between other infections and typhoid infections, with the exception of STY1522 (*p* < 0.05 Wilcoxon Rank sum). B) Boxplots of antibody responses against (in order) STY4539, STY1886, Vi. All mean antibody measurements are statistically significant between the healthy controls and typhoid infections and between other infections and typhoid infections (*p* < 0.05 Wilcoxon Rank sum).

IgM against all twelve of the protein antigens and the Vi polysaccharide was significantly elevated in the plasma of the typhoid patients in comparison with the healthy controls (*p* < 0.05) (Fig. 2). Furthermore, there was a significant differentiation in the plasma IgM measurements between the typhoid patients and those with a febrile disease with an alternative confirmed etiology with all antigens with the exception of STY1522 (*p* < 0.05).

By assessing raw antibody measurements, we surmised that the best performing antigens, with respect to differentiating between the patient groups were STY4539, STY1886, and Vi (Fig. 2). The mean IgM responses (OD values) in the afebrile controls, the other confirmed infections and the typhoid infections were 0.29, 0.27, and 0.42 (against STY4539), 0.17, 0.18, and 0.25 (against STY1886), and 0.22, 0.21, and 0.35 (against Vi), respectively. This segregation between the patient groups was highly significant, resulting in *p*-values of 0.0001 and 0.003 for IgM against STY4539; <0.0001 and 0.004 for IgM against STY1886; and 0.0001 and 0.0001 for IgM against Vi, between healthy controls and typhoid infections and between other febrile diseases and typhoid infections, respectively (Wilcoxon signed-rank test).

### Sensitivity and specificity of the serodiagnostic antigens

To further assess the IgM responses against the various *S*. Typhi antigens for the purposes of diagnostic testing we calculated sensitivities and specificities using a validation group incorporating two positive reference groups and a negative reference group for additional statistical power. The negative reference group (n = 57) was the combination of data from the afebrile controls (n = 40) and from those with a confirmed diagnosis other than typhoid (n = 17 cases). The assay results were validated independently with two sets of positive reference data; these were a combination of blood culture confirmed *S*. Typhi along with those with a positive PCR amplification result for *S*. Typhi from blood (n = 32), and the blood culture confirmed *S*. Typhi patients only (n = 19).

The IgM responses against each of the antigens generated a continuous data set that was used to generate ROC curves to optimize the index cut-off value. The defined cut-off values of the thirteen antigens corresponded with a range of sensitivities ranging from 0.50 to 0.84 and specificities between 0.58 and 0.84; areas under the ROC curve (AUC) ranged from 0.7 to 0.85. When used alone none of the antigens demonstrated a sensitivity or specificity >0.8. As predicted, Vi, STY4359, and STY1886 were the three antigens with the greatest serodiagnostic capacity in discriminating typhoid cases from afebrile controls and other infections. The sensitivities and specificities for identifying typhoid patients by IgM titers were 0.68; 0.8 (Vi), 0.62; 0.82 (STY4539), and 0.62; 0.82 (STY1886), respectively. Correspondingly, the AUCs were 0.84 (95%CI: 0.71, 0.96), 0.77 (95%CI: 0.61, 0.92), and 0.77 (95%CI: 0.62, 0.91).

**Figure 3:**
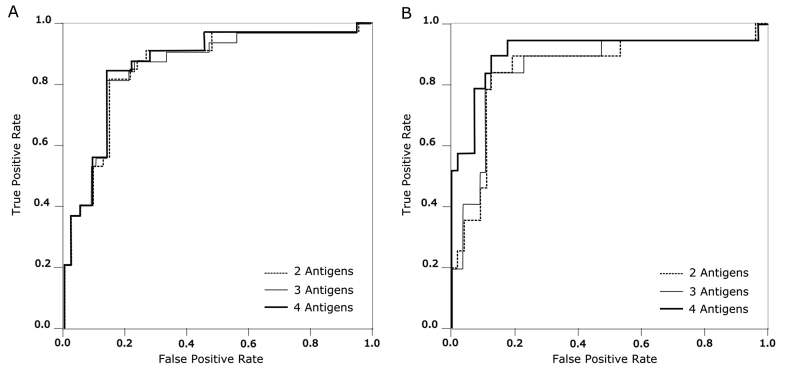
Assessing the sensitivity and specificity of IgM against *Salmonella* Typhi antigens for the diagnosis of typhoid fever. Receiver operating characteristic (ROC) curves summarizing the antibody responses against antigen combinations for the diagnosis of typhoid. The x-axis displays the false positivity rate (Specificity) and the y-axis displays true positive rate (Sensitivity). The performance of two, three and four antigens are shown by the dashed, gray, and black lines, respectively. A) ROC curve produced when the positive references are typhoid cases confirmed by blood culture and PCR amplification (n = 32). B) ROC curve produced when the positive references are typhoid cases confirmed by blood culture only (n = 19).

We next employed SVM to identify combinations of two or more antigens across all 13 antigens to increase overall sensitivity and specificity. Using confirmed *S*. Typhi infection by blood culture or PCR as the positive reference (n = 32), we found 11 combinations of two to four antigens that gave sensitivities from 0.81 to 0.84, specificities from 0.81 to 0.86, and AUCs from 0.87 to 0.87 (Fig. 3). We additionally identified 17 combinations of two to four antigens when using the positive reference as culture confirmed *S*. Typhi only (n = 19), obtaining sensitivities from 0.84 to 0.89, specificities from 0.88 to 0.94, and AUCs from 0.859 to 0.912 (Fig. 3, Table 2, Table 3).

**Table 2:** The sensitivity and specificity of multiple antigens for typhoid diagnosis using blood culture and PCR positive patients as positive reference group (n = 32).

Antigen combinations	STY1703 & Vi	STY4539 & STY1703 & Vi	STY4539 & STY1703 & STY1886 & Vi
Specificity	0.86	0.86	0.86
Sensitivity	0.81	0.84	0.84
Number with undiagnosed febrile disease predicted to have typhoid (n = 243)	135 (59%)	134 (59%)	134 (59%)
Number with clinically suspected typhoid disease predicted to have typhoid (n = 18)	16 (88%)	16 (88%)	16 (88%)
AUC (95%CI)	0.865 (0.782, 0.947)	0.863 (0.779, 0.947)	0.866 (0.783, 0.949)

**Table 3:** The sensitivity and specificity of multiple antigens for typhoid diagnosis using blood culture positive patients only as positive reference group (n = 19).

Antigen combinations	STY1886 & Vi	STY4190 & STY1886 & Vi	STY4539 & STY4190 & STY1886 & Vi
Specificity	0.88	0.89	0.88
Sensitivity	0.84	0.84	0.89
Number with undiagnosed febrile disease predicted to have typhoid (n = 226)	119 (52%)	78 (34%)	71 (31%)
Number with clinically suspected typhoid disease predicted to have typhoid (n = 18)	15 (83%)	13 (72%)	13 (72%)
AUC (95%CI)	0.859 (0.746, 0.972)	0.891 (0.791, 0.991)	0.912 (0.81, 1.014)

For the positive reference sets, IgM against Vi contributed to all of the combinations, while STY1703, STY1886, and STY4539 were present in more than half of the combinations. The remaining nine antigens contributed to at least one combination that gave sensitivities and specificities >0.8. These results demonstrated that, in the majority of examples, a combination of up to four antigens was directly associated with an increased performance of the IgM serology. However, the best performing antigens for the identification of typhoid patients by IgM were Vi in combination with either STY1703 or STY1886 (Table 2, Table 3).

### Identifying typhoid cases in patients with undiagnosed febrile disease

**Figure 4:**
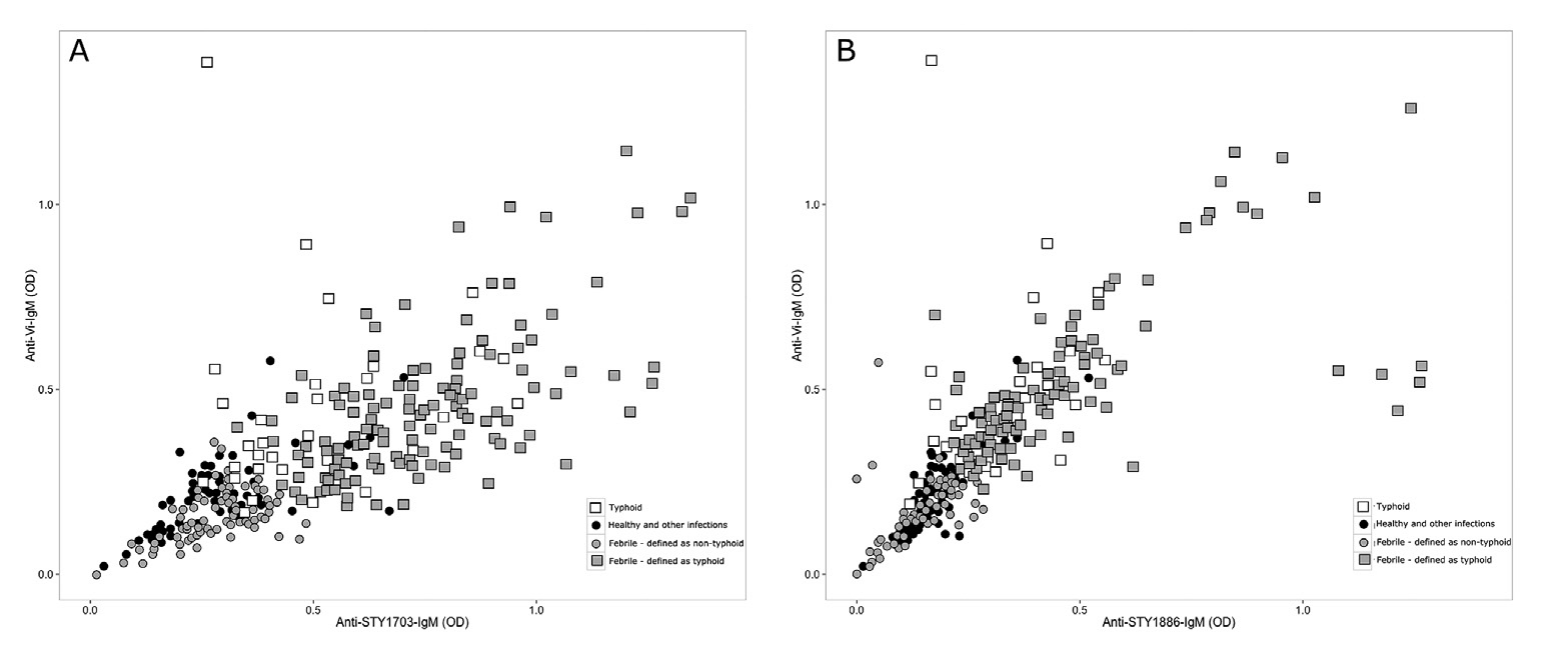
Detecting febrile patients with an IgM profile indicative of typhoid fever. Plots predicting the number of undiagnosed febrile patients that have an IgM measurement indicative of typhoid fever. The black circles represent the negative controls, which includes healthy controls and patients other with infections. The white boxes represent typhoid cases confirmed by blood culture or PCR. The gray circles are febrile patients with an IgM profile indicative of not having typhoid fever, gray boxes are febrile patients defined as having a typhoid infection using the pre-defined IgM profile. A) Plot where the positive reference was defined as the typhoid cases confirmed by blood culture or PCR (n = 32); the selected antigen combination was STY1703 and Vi. B) Plot where the positive reference was defined as the typhoid cases confirmed by blood culture only (n = 19); the selected antigen combination was STY1886 and Vi.

In this Bangladeshi cohort there were 226 patients with febrile disease without laboratory confirmed etiology and 18 with clinically suspected typhoid that were negative by blood culture and PCR amplification. We aimed to estimate the proportion of patients in this population who may have typhoid by applying the SVM cutoffs and combining the IgM titers against two *S*. Typhi antigens. We performed two independent analyses; the first combined IgM titers against STY1703 and Vi using a combination of culture confirmed *S*. Typhi and positive PCR amplification for *S*. Typhi from blood as the positive reference group (Fig. 4 and Table 3). Using these criteria we found that 142/226 (59%) of the undiagnosed febrile patient group and 16/18 (88%) with clinically suspected typhoid had IgM titers indicative of typhoid. Using more stringent criteria (blood culture confirmed patients) as the positive reference and a combination of IgM against Vi and STY1886 we found that 119/226 (52%) febrile cases and 15/18 (83%) clinically suspected typhoid, respectively, had a profile indicative of typhoid (Fig. 4).

## Discussion

Typhoid is caused by a human restricted pathogen that is transmitted fecal-orally. Consequently, improving the quality of drinking water supplies and education in better hygiene practices are likely the most effective measures to for typhoid elimination. However, these interventions cannot be promptly realized in the endemic areas of Africa and South Asia. Therefore, the short-term control of typhoid is dependent on large vaccination programmes and appropriate treatment, both of which, for differing reasons, rely on better case detection. The fact that there is not currently a typhoid diagnostic assay with a high degree of sensitivity or specificity limits disease burden assessments,^26^, ^27^ and may result in patients being misdiagnosed and receiving a sub-optimal therapy.^28^ Furthermore, with the global increase in *S*. Typhi associated with reduced susceptibility and resistance against the fluoroquinolones and other antimicrobials,^29^, ^30^ the demand for typhoid diagnostics are now greater than ever.^31^, ^32^

There is a paucity of data arising from studies on humans with typhoid that have measured the immunological response to *S*. Typhi specific antigens.^7^ Studies that have been performed have found significant interferon-γ (IFN-γ) responses in cells stimulated with various antigens including fimbriae and outer membrane proteins using polymorphonuclear cells from the blood of typhoid patients.^33^ Further, when whole blood from typhoid patients is stimulated with *S*. Typhi lipopolysaccharide, TNFα release is lower during active typhoid than after antimicrobial treatment, indicating a short immune modulation effect, which may be induced by the Vi polysaccharide.^34^ Antigen arrays and other probing techniques have being used successfully to interrogate the antibody repertoire during early infection,^22^, ^35^, ^36^, ^37^ and have detected antibody responses to several novel, and potentially organism specific, antigens that may be able to distinguish typhoid patients from controls.^22^ Using this “screening data” we rationally selected several *S*. Typhi protein antigens and aimed to investigate if early immunological diagnostic signals could be detected in the plasma of febrile patients.

Our study focused on a well-defined patient group from Bangladesh, who were enrolled for the primary focus of studying typhoid diagnostics. Whilst the patient numbers with typhoid in this group were relatively modest, the clinical and laboratory criteria for patients with febrile disease were consistent and have been previously assessed with commercial serological tests for typhoid.^23^ We noted that the IgM response against the twelve purified *S*. Typhi protein antigens were stable and well correlated; the IgM responses in comparison to Vi correlated less consistently. These data confirm our original antigen screening data and suggest that these antigens are immunogenic and induce an antibody response early in infection. Given the poor performance of commercial RDTs,^38^, ^39^ our data signify that the early detection of IgM against more specific *S*. Typhi protein antigens may be a more specific and sensitive approach for developing a RDT for typhoid.

Indistinguishable clinical features and the lack of a reliable gold standard test complicate typhoid diagnosis. Here, the IgM response against all 12 antigens was significantly higher in typhoid patients than both afebrile controls and patients with febrile diseases other than typhoid. Furthermore, through inference from the AUC under the ROC curve we were able to identify the best three performing antigens, which were encoded by STY4539 (PilL) and STY1886 (CdtB) in combination with the Vi polysaccharide. PilL is a putative exported protein and a component of the type IV pili encoded adjacent to the genes encoding Vi on SPI-7.^40^, ^41^ The PilL protein is induced following uptake by human derived macrophages,^42^ and the type IV pili to which it is associated facilitates entry into epithelial cells.^43^ CdtB, encoded by STY1886, is one of the two A sub-units of typhoid toxin, an AB type toxin.^44^ Typhoid toxin is a virulence-associated factor of *S*. Typhi, which is thought to be associated with the early symptoms of typhoid.^45^ We confirm that this component of typhoid toxin is immunogenic and may be an important biomarker of acute typhoid.^22^, ^46^ Whilst these three virulence factors (PilL, CdtB, and Vi) were not sufficient in themselves to produce a reproducibly high (>0.8) degree of sensitivity and specificity for typhoid diagnosis, we gained additional power by combining data from >1 antigen using an SVM model. The IgM responses against Vi in combination with either PilL or CdtB were found to generate the highest degree of sensitivity and specificity. Seemingly, a combination of the differing IgM responses against polysaccharide and a protein compensates for a lower affinity to one of the antigens. Furthermore, we were able to estimate the proportion of the population that may have typhoid by imposing cut-offs from the typhoid confirmed patients onto the population with undiagnosed febrile diseases. These data did not generate a precise cut-off, therefore, our data suggest that typhoid diagnostics are not an exact science and our data should be interpreted with caution. These methods warrant further investigation in additional cohorts, but it suggests a substantial burden of undiagnosed febrile disease is associated with *S*. Typhi in this setting.

This study has limitations. The sample size was relatively small; we aimed to rectify this by including a subset of patients that appear to have *S*. Typhi DNA in their bloodstream but were culture negative.^47^ Using this combination of methods as a gold standard we were able to increase the diagnostic power of the assays. A further limitation is that this study was conducted in a single healthcare location over a limited time period. Whilst our data provide some confidence that these serological assays may be of utility for typhoid diagnostics, these methods should to be validated in additional cohorts. However, there remains a challenge in identifying typhoid patients that have a sterile blood culture; a combination of novel approaches, such as metabolomics and/or functional genomics,^17^, ^21^ in a febrile disease cohort may add further insight into this important patient group.

In conclusion, we have investigated the serological diagnostic potential of *S*. Typhi protein antigens and the Vi polysaccharide in a group of patients with febrile diseases in Bangladesh. Our novel data show that serology may have some utility for typhoid diagnostics and a combination of antigens improves the diagnostic potential. Our assays give high levels of sensitivity and specificity, but require further assessment in differing patient populations.

## Conflict of interest

The authors declare no competing interests.

## Funding sources

This project was funded by the Wellcome Trust of Great Britain (106158/Z/14/Z) and the Bill and Melinda Gates Foundation. SB is a Sir Henry Dale Fellow, jointly funded by the Wellcome Trust and the Royal Society (100087/Z/12/Z). PTD is funded as a leadership fellow through the Oak Foundation. The funders had no role in study design, data collection and analysis, decision to publish, or preparation of the manuscript.

## Appendix A Supplementary data

Table S1 Plasmid constructs of *Salmonella* Typhi antigens in this study. Table S1
