# Supplementary material for: An evaluation of purified Salmonella Typhi protein antigens for the serological diagnosis of acute typhoid fever
Source: J Infect. 2017 Aug;75(2):104–14. doi: 10.1016/j.jinf.2017.05.007 (PMC5522525; doi:10.1016/j.jinf.2017.05.007)
Supplement: Table S1 — Plasmid constructs of Salmonella Typhi antigens in this study. [file mmc1.docx]

| Target | Construct name | 5'RE | Ty2 nomenclature and Coding sequence | 3'RE | Forward primer | Reverse primer | bp | aa | kDa |
| --- | --- | --- | --- | --- | --- | --- | --- | --- | --- |
| STY4190 | pEK90 | NcoI | 3904 (Typhi) aa25-495 | NotI | catg ccatgg gt  GATGCGCTCCAGCCCGATC | gcatgagc gcggccgc  CTGTGCCGCCGGTGTTTCC | 1413 | 471 | 52.6 |
| STY3208 | pEK91 | NcoI | 2970 (Typhi) aa1-279 | NotI | catg ccatgg gt  ATGGCAGCTAACGGAGAAAATAATCC | gcatgagc gcggccgc  CCAGGTCTTACCTATTTTAAATTCCC | 837 | 279 | 30.5 |
| STY1767 | pEK92 | NcoI | 1224 (Typhi) aa21-154 | NotI | catg ccatgg gt  GCACCGGCGCCAAATGCCAG | gcatgagc gcggccgc  AATTCGCCGTGCCTGCCAGAAG | 402 | 134 | 15.1 |
| STY1703 | pEK93 | NcoI | 1285 (Typhi) aa1-124 | NotI | catg ccatgg gt  ATGCGTATTACCAAAGTTGAGGG | gcatgagc gcggccgc TTCGCTATTCTTAACATAGAATATCTC | 372 | 124 | 14.1 |
| STY1522 | pEK94 | NcoI | 1459 (Typhi) aa25-363 | NotI | catg ccatgg gt TGCACAACCCTTGCTATTCAGGATAAAC | gcatgagc gcggccgc TCCTTTGACGTTGATTTTCTCGAACAC | 1017 | 339 | 37.6 |
| STY1886 | pEK95 | NcoI | 1111 (Typhi) aa28-269 | NotI | catg ccatgg gt  AAAGTTATGACCTGGAATCTTCAGGG | gcatgagc gcggccgc  ACAGCTTCGTGCCAAAAAGGCTAC | 726 | 242 | 26.4 |
| STY1498 | pEK96 | NcoI | 1477 (Typhi) aa203-305 | NotI | catg ccatgg gt  GGCGTGATTGAAGGGAAATTGATTCc | gcatgagc gcggccgc  GACGTCAGGAACCTCGAAAAGCG | 309 | 103 | 11.5 |
| STY3375 | pEK99 | NcoI | 3116 (Typhi) aa1-118 | NotI | catg ccatgg gt  ATGGCGTCCACATATCGCAC | gcatgagc gcggccgc  CTCTTGTGGATCGACTGGC | 354 | 118 | 13.8 |
| STY1372 | pEK100 | NcoI | 1594 (Typhi) aa27-74 | NotI | catg ccatgg gt  AGCAACCGCGCCGGTCGG | gcatgagc gcggccgc  GCGCTCTCTCCAGTTCGGATG | 144 | 48 | 5.7 |
| STY1612 | pEK101 | NcoI | 1376 (Typhi) aa30-108 | NotI | catg ccatgg gt  AGTAAAACAGAAGAACGCCAGGC | gcatgagc gcggccgc  TTGACCTCCGGTATTGCGGTAC | 237 | 79 | 9.2 |
| STY0357 | pEK102 | NcoI | 2538 (Typhi) aa20-246 | NotI | catg ccatgg gt  GGTTTGCTGAGCAGCAGCAGC | gcatgagc gcggccgc  TTTTGCCTCGGAGAGCGTATAATTTG | 681 | 227 | 25.8 |
| STY4539 | pEK103 | NcoI | 4239 (Typhi) aa20-414 | NotI | catg ccatgg gt  CAGCAGACCTCCACCCAAACC | gcatgagc gcggccgc  CGACCGGTCAGCCGGTTTATC | 1185 | 395 | 41.9 |
| STY0452 | pEK104 | NcoI | 2449 (Typhi) aa22-179 | NotI | catg ccatgg gt  CCGCAGAGCGAAGTTCGC | gcatgagc gcggccgc  TTGATTAACAGGCTGAATATCATGG | 474 | 158 | 17.2 |
| STY0796 | pEK105 | NcoI | 2126 (Typhi) aa27-262 | NotI | catg ccatgg gt  CAGGCGCCAATCAGTAGTGTC | gcatgagc gcggccgc  CATCGCGTTAAGACGCTTCTGC | 708 | 236 | 25.4 |
| STY0065 | pEK106 | NcoI | 0058 (Typhi) aa33-79 | NotI | catg ccatgg gt  GGTATGTCTGCCGTGATTACTC | gcatgagc gcggccgc  AGCGTTAAGGCGGTGATGGTG | 138 | 46 | 4.9 |
| STY3093 | pEK107 | NcoI | 2864 (Typhi) aa1-118 | NotI | catg ccatgg gt  ATGTTTTTGACGTACATTTCATTTCagg | gcatgagc gcggccgc  CTGGCCTTTGGCGTTAATTTTAC | 354 | 118 | 13.6 |
| STY1086 | pEK108 | PciI | 1855 (Typhi) aa20-178 | NotI | catg acatgt ta  GGAGAAAATAAAAGCTATTATCAGCTC | gcatgagc gcggccgc  AGCGGCCGCTTCCTGACTC | 477 | 159 | 17.4 |
| STY3765 | pEK109 | NcoI | 3515 (Typhi) aa1-518 | NotI | catg ccatgg gt  ATGAAAGTAAAACTGCTTGCTGCC | gcatgagc gcggccgc  CTTCTTCACATCCGCAACACG | 1554 | 518 | 55.5 |

**Table S1.** Plasmid constructs of *Salmonella* Typhi antigens generated in this study
